# Tracing the origins of midlife despair: association of psychopathology during adolescence with a syndrome of despair-related maladies at midlife

**DOI:** 10.1017/S0033291723001320

**Published:** 2023-12

**Authors:** Grace M. Brennan, Terrie E. Moffitt, Antony Ambler, HonaLee Harrington, Sean Hogan, Renate M. Houts, Ramakrishnan Mani, Richie Poulton, Sandhya Ramrakha, Avshalom Caspi

**Affiliations:** 1 Duke Aging Center, Duke University School of Medicine, Durham, NC, USA; 2 Department of Psychology & Neuroscience, Duke University, Durham, NC, USA; 3 Department of Psychiatry and Behavioral Sciences, Duke University School of Medicine, Durham, NC, USA; 4 Center for the Study of Population Health and Aging, Duke University Population Research Institute, Durham, NC, USA; 5 Institute of Psychiatry, King's College London, London, UK; 6 Center for Genomic and Computational Biology, Duke University, Durham, NC, USA; 7 Promenta, University of Oslo, Oslo, Norway; 8 Department of Psychology and Dunedin Multidisciplinary Health and Development Research Unit, University of Otago, Dunedin, New Zealand; 9 School of Physiotherapy, University of Otago, Dunedin, New Zealand

**Keywords:** Adolescent psychopathology, deaths of despair, lifespan development, mental health, midlife, pain, risk factors, sleep problems, substance misuse, suicidality

## Abstract

**Background:**

Midlife adults are experiencing a crisis of deaths of despair (i.e. deaths from suicide, drug overdose, and alcohol-related liver disease). We tested the hypothesis that a syndrome of despair-related maladies at midlife is preceded by psychopathology during adolescence.

**Methods:**

Participants are members of a representative cohort of 1037 individuals born in Dunedin, New Zealand in 1972–73 and followed to age 45 years, with 94% retention. Adolescent mental disorders were assessed in three diagnostic assessments at ages 11, 13, and 15 years. Indicators of despair-related maladies across four domains – suicidality, substance misuse, sleep problems, and pain – were assessed at age 45 using multi-modal measures including self-report, informant-report, and national register data.

**Results:**

We identified and validated a syndrome of despair-related maladies at midlife involving suicidality, substance misuse, sleep problems, and pain. Adults who exhibited a more severe syndrome of despair-related maladies at midlife tended to have had early-onset emotional and behavioral disorders [*β* = 0.23, 95% CI (0.16–0.30), *p* < 0.001], even after adjusting for sex, childhood SES, and childhood IQ. A more pronounced midlife despair syndrome was observed among adults who, as adolescents, were diagnosed with a greater number of mental disorders [*β* = 0.26, 95% CI (0.19–0.33), *p* < 0.001]. Tests of diagnostic specificity revealed that associations generalized across different adolescent mental disorders.

**Conclusions:**

Midlife adults who exhibited a more severe syndrome of despair-related maladies tended to have had psychopathology as adolescents. Prevention and treatment of adolescent psychopathology may mitigate despair-related maladies at midlife and ultimately reduce deaths of despair.

## Introduction

Deaths of despair (i.e. deaths from suicide, drug overdose, and alcohol-related liver disease; Case & Deaton, 2017) are rising among working-age adults in the U.S. (Case & Deaton, 2015; National Academies of Sciences, 2021; Shiels et al., 2020; Woolf & Schoomaker, 2019) and possibly other countries as well (Allik, Brown, Dundas, & Leyland, 2020; Augarde, Gunnell, Mars, & Hickman, 2022; Brown, Allik, Dundas, & Leyland, 2019; Probst & Rehm, 2018; Walsh et al., 2021). In the U.S., these increases are commonly conflated with the opioid epidemic and its concomitant misprescription of opioids (National Academies of Sciences, 2021; Szalavitz, 2021). However, deaths of despair and related maladies (e.g. suicidality, substance misuse) can proliferate anywhere, given certain societal conditions (e.g. inequality, political and economic shifts, lack of communal support; Blakely, Tobias, & Atkinson, 2008; Brainerd, 2021; King, Scheiring, & Nosrati, 2022; Sterling & Platt, 2022). Moreover, relatively few studies have examined despair-related maladies outside of the U.S. context. Here, we adopt a lifespan developmental approach to examine the characteristics of midlife adults afflicted by a syndrome of despair-related maladies. In a population-representative cohort of New Zealanders who have been followed from birth to midlife, we tested: (1) a hypothesized model of a multi-faceted despair syndrome at midlife, encompassing not only suicidality and substance misuse, but also sleep problems and pain; and (2) the hypothesis that adults with a more severe despair syndrome at midlife tended to have had psychopathology in adolescence.

The hypothesis that there is a multi-faceted midlife despair syndrome consisting of a constellation of maladies (i.e. suicidality, substance misuse, sleep problems, and pain) is based on evidence that these maladies rarely exist in isolation (Driscoll, Edwards, Becker, Kaptchuk, & Kerns, 2021; Oquendo & Volkow, 2018; Turecki et al., 2019). Although the contributions of suicidality and substance misuse to deaths of despair are clear and well recognized, two additional maladies that are born out of the life experiences of despair-afflicted adults are sleep problems and musculoskeletal pain (Case & Deaton, 2021; Case, Deaton, & Stone, 2020; Drake, Roehrs, Richardson, Walsh, & Roth, 2004; Hartvigsen et al., 2018). Lower educational attainment (i.e. not having a bachelor's degree) appears to be the strongest demographic predictor of deaths of despair (Case & Deaton, 2021). Individuals without a bachelor's degree are more likely to hold jobs characterized by difficult working conditions that can directly cause sleep problems and pain (Drake et al., 2004; Matre et al., 2021). Indeed, media accounts of deaths of despair portray lives in which deaths from illicit substance use followed from medications initially prescribed to treat the sleep problems and pain that arose from difficult working conditions (e.g. night or shift work, heavy physical labor).

Suicidality, substance misuse, sleep problems, and pain not only frequently co-occur; they also exacerbate one another. For example, individuals may be prescribed sedatives and opioids to address sleep problems and pain, which in turn increases their risk for substance misuse (Koffel, DeRonne, & Hawkins, 2020). Similarly, substance misuse is one of the strongest risk factors for suicide (Lynch et al., 2020). Moreover, since sleep problems and musculoskeletal pain indicate the presence of psychic and physical suffering that individuals often seek to quiet or end, it seems reasonable that these maladies might exist as part of a syndrome that can be identified before an individual succumbs to a death of despair. Taken together, extant research suggests the existence of a multi-faceted despair syndrome; it also implies that examining despair-related maladies individually provides a limited perspective that underestimates individuals' true risk for dying a death of despair. Yet previous research tends to study these maladies in isolation, and in clinical practice they are commonly treated individually in separate clinics.

Since midlife despair is postulated to form a syndrome of interconnected maladies, it stands to reason that this syndrome has an antecedent in early life. In pursuit of this antecedent, we propose that psychopathology in adolescence represents a promising candidate factor for several reasons. First, the onset of psychopathology is most likely to occur during adolescence (Kessler et al., 2005; Moffitt & Caspi, 2019; Polanczyk, Salum, Sugaya, Caye, & Rohde, 2015). Second, psychopathology in adolescence disrupts the transition from school to work life (Bardone, Moffitt, Caspi, Dickson, & Silva, 1996; Hale, Bevilacqua, & Viner, 2015; Veldman et al., 2014) and, third, predicts poor aging-related outcomes (Bourassa et al., 2022). We hypothesized that adolescent psychopathology could be a starting point for a syndrome of despair-related maladies because it is known to disrupt educational attainment, limiting individuals' employment opportunities and making them more likely to have jobs characterized by difficult working conditions. Finally, psychopathology in adolescence is treatable and thus amenable to prevention efforts (Deković et al., 2011; Lee, Horvath, & Hunsley, 2013; Swan et al., 2018). Identifying a modifiable risk factor for a syndrome of midlife despair would allow prevention and intervention strategies to focus on vulnerable populations early in development to prevent later deaths of despair. However, despite the need to understand developmental pathways to despair (Shanahan & Copeland, 2021), previous research has not yet examined whether adolescent psychopathology precedes a syndrome of despair-related maladies in midlife, a time in the lifecourse during which deaths of despair were first identified (Case & Deaton, 2015) and during which mortality rates have been rising most steeply for decades (Case & Deaton, 2017; National Academies of Sciences, 2021; Tilstra, Simon, & Masters, 2021). Converging evidence across high-income nations suggests that despair-related maladies may peak during midlife (Case et al., 2020; Giuntella et al., 2022).

The present study fills three major gaps. First, we examine associations between individual-level characteristics and a midlife despair syndrome, in contrast with previous research that has focused on trends at the population level. Second, we adopt a lifespan developmental perspective for studying midlife despair-related maladies, looking back several decades in individuals' lives, in contrast with previous research that has provided point-in-time descriptions of rates of despair-related maladies and deaths. A developmental perspective is needed because knowledge about individuals' personal histories can help inform prevention strategies. Finally, we take a comprehensive and cross-domain approach to measuring a midlife despair syndrome, in contrast with previous research that has examined despair-related maladies individually (Copeland et al., 2020).

The present study is well suited to examine adolescent psychopathology as an antecedent of a midlife despair syndrome due to its prospective longitudinal design and multi-modal (i.e. self-report, informant-report, and national register data) assessment of midlife despair-related maladies. We used confirmatory factor analysis (CFA) to test our hypothesized model of a midlife despair syndrome using 12 indicators measured when participants were 45 years old. We tested the construct validity of the midlife despair syndrome by examining associations with putatively related external constructs, including lower educational attainment, difficult working conditions, social disconnection, diminished wellbeing, and pessimism about the future. We then tested whether adults with more despair-related maladies at midlife tended to have had a mental disorder [i.e. depression, an anxiety disorder, attention-deficit/hyperactivity disorder (ADHD), or conduct disorder] in adolescence. Because sex, childhood socioeconomic status (SES), and childhood IQ are known risk factors for individual despair-related maladies (Bartley & Fillingim, 2013; McHugh, Votaw, Sugarman, & Greenfield, 2018; McLean et al., 2014; Nock et al., 2008; Turecki et al., 2019), we accounted for these potential confounding factors in our analyses. Additionally, we tested whether adults who had more despair-related maladies at midlife had more comorbid mental disorders in adolescence. Finally, although the term ‘despair’ implies that depression is the most relevant mental disorder for deaths of despair, we tested associations for each adolescent mental disorder individually to ascertain the generality *v.* specificity of associations.

## Methods

### Sample

Participants were members of the Dunedin Longitudinal Study, a representative birth cohort (*N* = 1037; 91% of eligible births; 51.6% male) born between April 1972 and March 1973 in Dunedin, New Zealand, who were eligible based on residence in the province and who participated in the first assessment at age 3 (Poulton, Moffitt, & Silva, 2015). The cohort represented the full range of SES in the general population of New Zealand's South Island and, in adulthood, matched the NZ National Health & Nutrition Survey on key health indicators (BMI, smoking, physical activity, doctor visits; Poulton et al., 2015) and the NZ Census on educational attainment (Richmond-Rakerd et al., 2020). The cohort consists primarily of White participants (93%), matching South Island demographics (Poulton et al., 2015). Assessments were carried out at birth and ages 3, 5, 7, 9, 11, 13, 15, 18, 21, 26, 32, 38, and, most recently (completed April 2019), 45 years. Overall, of the original 1037 cohort participants, 997 were still alive at age 45; of these, 938 (94.1%) participated in the age-45 assessments [474 men (50.5%)]. Participants assessed at age 45 did not differ significantly from other living participants in terms of childhood SES, childhood IQ, or history of psychopathology (online Supplementary eAppendix 1). Based on these analyses, data were assumed to be missing at random. Of the 938 who participated at age 45, 926 had sufficient data across indicators of despair-related maladies for inclusion in analyses (i.e. at least 50% of data points present within each indicator or subfactor).

The Dunedin Study was approved by the NZ-HDEC (Health and Disability Ethics Committee). The study protocol was approved by the institutional ethical review boards of the participating universities. Study members gave written informed consent before participating in each assessment.

### Assessment of adolescent psychopathology

Mental disorders [i.e. depression, an anxiety disorder, attention-deficit/hyperactivity disorder (ADHD), and conduct disorder] were assessed at ages 11, 13, and 15 (Table 1). Since adolescence is generally understood to be the transition phase between childhood and adulthood (World Health Organization, 2023), we considered ages 11 to 15 to represent adolescence in this sample. Age 18 was not included because students in New Zealand are able to leave school at age 15; thus, some participants were already in the labor force and living independently by age 18. Prevalence of psychopathology (Caspi et al., 2014) was comparable to that in U.S. epidemiological samples (Merikangas et al., 2010).

**Table 1.** Descriptions and descriptive statistics for adolescent psychopathology, other risk factors, and observed indicators of midlife despair-related maladies

Variable	Description	*Total N*	*M* (s.d.)/*N* (%)
Adolescent psychopathology	At ages 11, 13, and 15, the Diagnostic Interview Schedule for Children (Costello, Edelbrock, Kalas, Kessler, & Klaric, 1982), following the then-current DSM-III criteria, was used to assess adolescent psychopathology. Participants were defined as having adolescent psychopathology if they met full diagnostic criteria for depression, an anxiety disorder, ADHD, or conduct disorder between ages 11 and 15. Age 18 was not included because some participants were already in the labor force by age 18, and we wanted to capture the period during which all participants were still in school. Adolescent mental disorder comorbidity was operationalized as the number of mental disorders for which participants' symptoms met diagnostic criteria between ages 11 and 15 (possible range: 0–4; groups with 3 and 4 diagnoses were then combined because of small *N*s within each group; Fig. 3).	892	308 (34.5%)
Other risk factors			
Male sex	Participants' sex was coded as male (1) or female (0).	926	464 (50.1%)
Childhood SES	SES of participants' families during childhood was measured using a 6-point scale that assessed parents' occupational statuses, defined based on average income and educational levels derived from the New Zealand Census (Poulton et al., 2002). The higher occupational status of either parent was averaged across the childhood assessments at ages 3, 5, 7, 9, 11, 13, and 15 years.	921	3.78 (1.13)
Childhood IQ	The Wechsler Intelligence Scale for Children–Revised (WISC–R) (Wechsler, 1974) was administered to study members at ages 7, 9, and 11 years, and scores were averaged across these assessments. The test was individually administered on each occasion according to standard protocol. Psychometrists were blind to the children's performance on previous administrations of the WISC–R.	914	100.73 (13.82)
*Observed indicators of despair-related maladies*			
Suicidality			
Suicide attempted	In assessments carried out at ages 26, 32, 38, and 45 years, suicide attempts were queried during structured interviews about self-harm and suicide and again as a symptom of depression during structured diagnostic interviews for depression. Queries invoked 11 types of suicidal behaviors, including cutting or stabbing oneself, overdosing on pills, and attempting to hang or strangle oneself (Goldman-Mellor et al., 2014). Interviewers differentiated between suicide attempts and nonsuicidal self-harm; here we study incidents accompanied by self-reported intent to die. Life History Calendar interviews (Caspi et al., 1996) were used to ascertain the timing of suicide attempts. Study members were then coded as having attempted suicide (1) or not (0) between the ages of 26 and 45.^a^	926	61 (6.6%)
Treatment for suicidality	At age 45 years, Life History Calendar interviews were used to ascertain whether Study members received treatment for suicidality (1) or not (0) during the time since their last assessment at age 38. Treatment included inpatient treatment, outpatient treatment, or taking prescribed psychiatric medication.	925	7 (0.8%)
Informant-reported suicidality	At age 45 years, participants nominated 3 people ‘who know them well.’ The informants were mailed questionnaires and asked to complete a checklist, including the extent to which the participant ‘talks about suicide’ (0 = not a problem, 1 = a bit of a problem, 2 = definite problem). The returned informant ratings were then averaged for each participant (range: 0–2). Response rates were high at age 45 (94% of participants had at least one informant return the questionnaire, 88% had two or more returned, and 68% had all three).	883	0.04 (0.18)
Substance misuse			
Substance use disorder (SUD) symptoms	At age 45 years, participants were interviewed about their past-year use of alcohol, cannabis, and other illicit drugs via the Diagnostic Interview Schedule (Robins, Cottler, Bucholz, and Compton, 1995; Robins, Helzer, Croughan, and Ratcliff, 1981). The number of Diagnostic and Statistical Manual of Mental Disorders (DSM) criteria met for each substance category were then summed to derive the SUD symptoms variable (range: 0–30), with higher scores reflecting more severe substance misuse.	924	1.40 (2.53)
Treatment for substance misuse	At age 45 years, Life History Calendar interviews were used to ascertain whether participants received treatment for alcohol or other substance use during the time since their last assessment at age 38. Treatment included inpatient treatment, outpatient treatment, or taking prescribed psychiatric medication. Treatment receipt (0 = no, 1 = yes) for each of the two substance categories (i.e. alcohol, other substances) was then summed to derive the substance misuse treatment variable (range: 0–2).	925	0.06 (0.27)
Informant-reported substance misuse	As part of the questionnaires mailed to informants at age 45 years (see informant-reported suicidality, above), informants rated two items related to substance misuse: (1) whether the participant has marijuana/drug problems and (2) whether the participant has alcohol problems (0 = not a problem, 1 = a bit of a problem, 2 = definite problem). Scores were summed to derive the informant-reported substance misuse variable (range: 0–4), with higher scores reflecting perceptions of more severe substance misuse.	883	0.29 (0.59)
Sleep problems			
Pittsburgh Sleep Quality Index (PSQI)	At age 45 years, the PSQI was used to assess (poor) sleep quality (Buysse, Reynolds, Monk, Berman, and Kupfer, 1989). The PSQI consists of 18 self-report items relating to sleep patterns and different forms of sleep impairment in the past month. These questions are used to derive scores for seven different components of sleep (subjective sleep quality, sleep latency, sleep duration, habitual sleep efficiency, sleep disturbances, use of sleep medication, and daytime dysfunction), each scored from 0 to 3. These scores were summed to derive the PSQI global sleep quality variable (range: 0–21), with higher scores reflecting poorer sleep quality.	909	6.22 (2.43)
Sleep aid use	At age 45 years, participants reported how often they used medicine (prescribed or over-the-counter), alcohol, or cannabis to help them sleep in the past month (0 = not during the past month, 1 = occasionally, 2 = regularly).	915	0.37 (0.68)
Social jetlag	At age 45 years, participants reported on their level of social jetlag, or the discrepancy between their internal clock (i.e. circadian rhythm) and external schedule. Social jetlag was calculated by subtracting each participant's midpoint of sleep on work days from their midpoint of sleep on free days. Outliers were recoded, and all analyses were conducted using the absolute value of social jetlag (see Parsons et al., 2015).	901	0.84 (0.85)
Pain			
Musculoskeletal pain composite	At age 45 years, participants self-reported whether they experienced pain in their muscles, bones, or joints lasting at least one week in the past 12 months. Participants used a body pain map to identify the number of sites where they experienced pain. Responses were categorized as no pain (0), single painful site (1), or multiple painful sites (2). Participants who endorsed pain then reported whether the pain caused them to take time off from work (0 = no, 1 = yes), receive or apply for disability or workers' compensation benefits (0 = no, 1 = yes), or consult a health professional (0 = no, 1 = yes) in the past 12 months. Scores were summed to derive the pain composite variable (range: 0–5), with higher scores reflecting more severe and debilitating pain.	908	1.68 (1.38)
Life spheres with musculoskeletal pain interference	At age 45 years, participants answered questions from the PROMIS Pain Interference scale (Amtmann et al., 2010) regarding the extent to which musculoskeletal pain interfered with the following five life spheres in the past 12 months: work activities, social activities, household chores, fun activities, and family life (0 = not at all, 4 = very much). The number of spheres in which participants reported significant musculoskeletal pain interference (i.e. responses of ‘quite a bit’ or ‘very much’) were summed (range: 0–5), with higher scores reflecting more life spheres negatively impacted by musculoskeletal pain.	908	0.37 (1.00)
Pain medication use	At age 45 years, we linked participants to their data in the New Zealand nationwide Pharmaceutical Collection, which holds information about prescription drugs filled by pharmacists. Data were coded according to whether the participant had been prescribed a pain medication (eg, opioids, NSAIDs) in the past year (0 = no, 1 = yes). Participants who did not reside in New Zealand in the past year were coded as using pain medication if (1) they reported taking any pain medication over the 14 days prior to the age-45 assessment day, and/or (2) the prescription containers that they were asked to bring with them to the general medical examination on the age-45 assessment day included pain medication.	727	270 (37.1%)

*Note.* Total *N* refers to the number of participants for whom data was available for each variable, whereas *N* in the farthest right column refers to the number of participants who had a score of ‘1’ for each dichotomous variable.

a Suicidal ideation was assessed within the depression interview, only if a participant endorsed at least one of the two gate items (i.e. depressed mood, anhedonia); therefore, suicidal ideation data were not available for a large portion of the sample and were not used in analyses.

### Measuring other potential risk factors

We selected other potential risk factors as covariates in regression models based on theory and documented associations with adolescent psychopathology and with suicidality, substance misuse, sleep problems, and pain: sex, childhood SES, and childhood IQ (Table 1).

### Measuring indicators of midlife despair-related maladies

We assessed 12 indicators of despair-related maladies at age 45 in four domains: suicidality, substance misuse, sleep problems, and pain (Table 1). Indicators were derived from a variety of modalities, including self-report (e.g. sleep quality), informant-report (e.g. perceived misuse of alcohol and drugs), and national register data (e.g. pain medication use). These indicators were used as observed variables in the CFA models. Although we could have included additional variables in the models, we restricted the models to the variables and domains listed in Table 1 for conceptual reasons. First, based on previous research, it is not clear that other constructs (i.e. depression) are truly part of the midlife despair syndrome. Instead, depression is typically conceptualized as an antecedent or cause of despair-related maladies (Copeland et al., 2020). Second, to avoid overlap between the midlife despair syndrome and adolescent psychopathology, we did not include depression and anxiety in the CFA models. However, validation analyses tested associations between the midlife despair syndrome and potentially related constructs at midlife (online Supplementary eAppendix 3).

### Statistical analysis

We used CFA to model the structure of midlife despair-related maladies. CFAs were performed using the weighted least squares means and variance adjusted (WLSMV) algorithm and 1000 bootstrapped samples in Mplus (version 8.7; Muthén and Muthén, 1998–2017). The WLSMV estimator is appropriate for categorical and nonmultivariate normal data and provides consistent estimates when data are missing at random with respect to covariates (Asparouhov & Muthén, 2010). The following variables were treated as categorical in analyses: suicide attempted, suicidality treatment, substance misuse treatment, sleep aid use, and pain medication use (see online Supplementary eAppendix 2 for more information regarding CFA). We then extracted the factor scores derived from CFA that represented the despair syndrome as a whole, as well as each of its constituent elements (i.e. suicidality, substance misuse, sleep problems, and pain), for use in further regression analyses.

To achieve our aim of ‘looking back’ into the developmental histories of participants afflicted with despair-related maladies, we conducted a series of regression analyses using adolescent psychopathology variables as predictor (independent) variables and midlife despair variables as outcome (dependent) variables. First, we performed ordinary least squares (OLS) regression to test whether adolescent psychopathology predicted the despair syndrome (i.e. the general despair factor derived from CFA) at midlife. To test whether sex, childhood SES, or childhood IQ could explain this association, we then adjusted for these risk factors. We also performed OLS regression to test whether the number of mental disorders diagnosed in adolescence predicted the midlife despair syndrome. Finally, we performed OLS regression to test whether specific adolescent disorders predicted the midlife despair syndrome.

The premise and analysis plan for this project were preregistered at https://dunedinstudy.otago.ac.nz/files/1652150270_Brennan_Concept%20Paper_Diseases%20of%20Despair_2-21-22.pdf. Results reported here were checked for reproducibility by an independent data analyst, who recreated the code by working from the manuscript and applying it to a fresh copy of the dataset.

## Results

### A measurement model of despair-related maladies at midlife

**Fig. 1.**
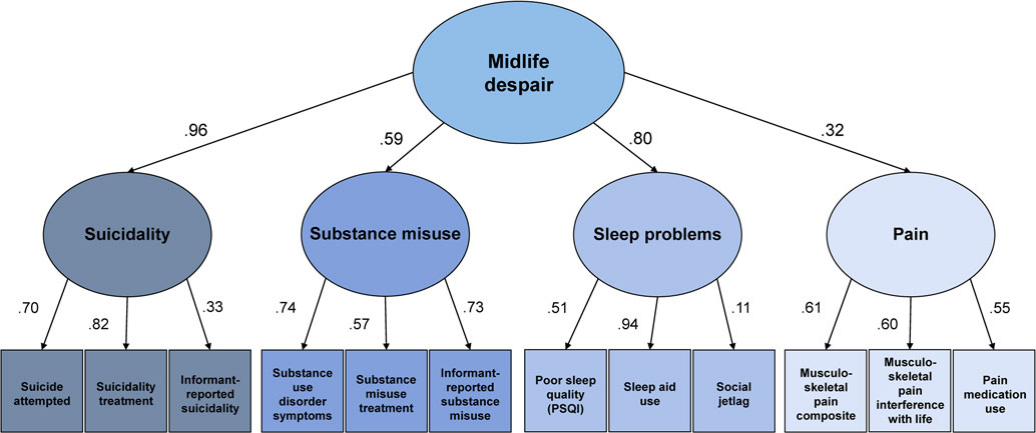
The structure of midlife despair-related maladies. *Note:* Ovals are latent (unobserved) factors representing a syndrome of midlife despair and its constituent elements; boxes are observed indicators of each constituent element. Numbers are standardized factor loadings.

CFA indicated that the higher-order factor model (Fig. 1) fit the data closely: CFI = 0.96, TLI = 0.95, RMSEA = 0.04, 90% CI [0.03–0.05], SRMR = 0.07 (online Supplementary eTable 1). Loadings for the suicidality, substance misuse, sleep problems, and pain subfactors on the general midlife despair factor were all significant and positive, ranging from moderate (0.32 for pain) to strong (0.96 for suicidality). Loadings for all of the indicators on their respective subfactors were significant and positive, ranging from relatively weak (0.11 for social jetlag) to strong (0.94 for sleep aid use; see online Supplementary eTable 2 for correlations between the observed indicators of midlife despair-related maladies).

### Validation of the general despair factor

The general midlife despair factor is hypothesized to reflect an underlying sense of despair; hopelessness about one's own future; social disconnection; and a lack of opportunity, both real and perceived. Analyses were performed to test the construct validity of the general midlife despair factor (see online Supplementary eAppendix 3 for full results). Consistent with expectations, participants with higher scores on the general midlife despair factor reported lower levels of satisfaction with their lives (*r* = −0.38, *p* < 0.001), greater pessimism toward growing older (*t* = 4.70, *p* < 0.001), and poorer self-rated health (*r* = −0.28, *p* < 0.001). They were less likely to believe they would live to age 75 (*t* = 6.20, *p* < 0.001). They were more likely to have experienced depression within the past year (*t* = −9.93, *p* < 0.001). They had weaker social connections, reporting more loneliness (*r* = 0.26, *p* < 0.001) and less social support (*r* = −0.21, *p* < 0.001). They reported being in less secure financial situations (*r* = −0.30, *p* < 0.001). They reported higher levels of perceived stress overall (*r* = 0.37, *p* < 0.001) and endorsed using more avoidant, short-term-oriented, and self-destructive strategies to cope with relationship and financial stress, including smoking more (*t* = −8.74, *p* < 0.001) and drinking more (*t* = −5.64, *p* < 0.001). They were less likely to have a bachelor's degree (*t* = 4.52, *p* < 0.001) and held lower-status jobs (*r* = −0.18, *p* < 0.001). Their jobs were more physically demanding (*r* = 0.20, *p* < 0.001) and more likely to cause pain and fatigue (*r* = 0.23, *p* < 0.001). They were more likely to believe that they would soon be physically unable to continue working at their current jobs (*t* = −5.04, *p* < 0.001). They were more likely to work night shifts (*t* = −3.07, *p* = 0.002). Overall, the picture that emerged from the nomological network of the despair factor was consistent with existing conceptualizations of individuals who are most vulnerable to deaths of despair (Case & Deaton, 2021).

### Associations between adolescent psychopathology and the midlife despair syndrome

**Fig. 2.**
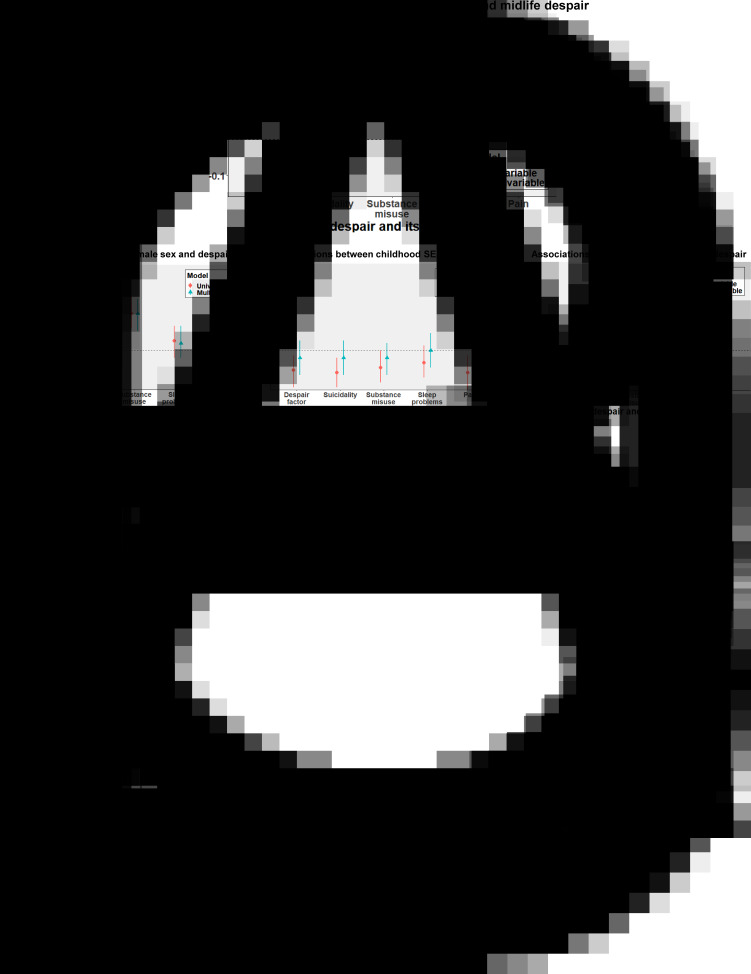
Adolescent psychopathology is associated with a syndrome of midlife despair and its constituent elements, even after adjusting for sex, childhood SES, and childhood IQ. *Note:* Panel A shows the associations between adolescent psychopathology and the despair factor at midlife, Panel B shows the associations between male sex and the midlife despair factor, Panel C shows the associations between childhood SES and the midlife despair factor, and Panel D shows the associations between childhood IQ and the midlife despair factor. Each panel depicts the results for univariable (simple) linear regression models including each risk factor as a single predictor, as well as results for multivariable (multiple) linear regression models including all risk factors as simultaneous predictors. Points and their associated vertical lines represent standardized effect sizes (*β*) and 95% confidence intervals, respectively. See also eTable 3.

Participants with psychopathology in adolescence had a more severe syndrome of despair-related maladies overall [*β* = 0.23, 95% CI (0.16–0.30), *p* < 0.001] and individually – suicidality [*β* = 0.24, 95% CI (0.17–0.31), *p* < 0.001], substance misuse [*β* = 0.19, 95% CI (0.12–0.27), *p* < 0.001], sleep problems [*β* = 0.19, 95% CI (0.12–0.26), *p* < 0.001], and pain [*β* = 0.18, 95% CI (0.11–0.25), *p* < 0.001], after adjusting for sex, childhood SES, and childhood IQ (Fig. 2, Panel A; see online Supplementary eTables 3 and 4 for adjusted and unadjusted estimates).

Figure 2, Panels B–D show the associations between sex, childhood SES, and childhood IQ and the midlife despair syndrome (see also online Supplementary eTable 3). Participants who were male, grew up in more deprived socioeconomic circumstances, and had lower IQ scores had more despair-related maladies overall at midlife. However, comparison of effect sizes indicated that associations between adolescent psychopathology and the midlife despair syndrome were generally several times as strong as associations for other risk factors; for example, adolescent psychopathology predicted the midlife despair syndrome 3–4 times more strongly than male sex, lower SES, and lower IQ. Moreover, the effects of more deprived socioeconomic circumstances and lower IQ were no longer significant in the multivariable models.

We also tested whether sex, childhood SES, or childhood IQ moderated the associations between adolescent psychopathology and despair-related maladies overall. We detected no interactions between any of these potential risk factors and adolescent psychopathology in the prediction of despair-related maladies overall (see online Supplementary eTable 5). Sensitivity analyses that used latent factors within the structural equation modeling framework (instead of extracted factor scores; online Supplementary eAppendix 4) and that included 6 additional participants who had died by suicide between ages 26 and 45 (online Supplementary eAppendix 5) yielded virtually indistinguishable results.

### Associations between number of mental disorders diagnosed in adolescence and the midlife despair syndrome

**Fig. 3.**
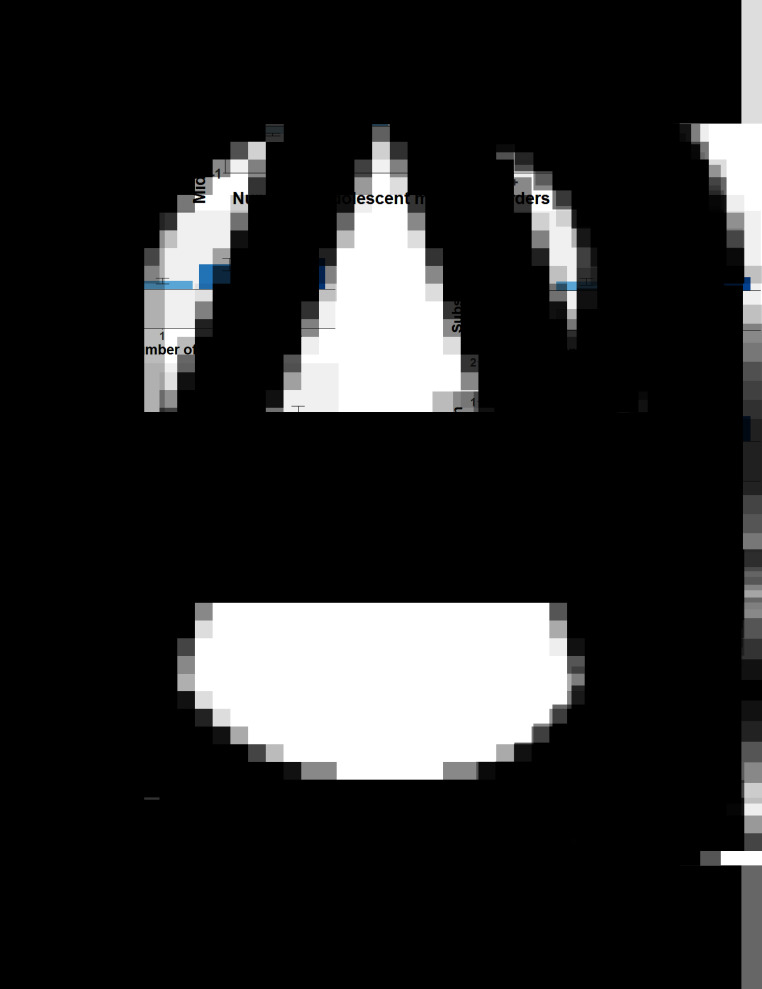
Dose-response associations between number of adolescent mental disorders and the midlife despair syndrome. *Note:* Panel A shows the association between number of adolescent mental disorders and the despair factor at midlife (*z*-scored), Panel B shows the association between number of adolescent mental disorders and the suicidality subfactor at midlife (*z*-scored), Panel C shows the association between number of adolescent mental disorders and the substance misuse subfactor at midlife (*z*-scored), Panel D shows the association between number of adolescent mental disorders and the sleep problems subfactor at midlife (*z*-scored), and Panel E shows the association between number of adolescent mental disorders and the pain subfactor at midlife (*z*-scored). Vertical lines represent ±1 standard error. 0 disorders, *N* = 580; 1 disorder, *N* = 212; 2 disorders, *N* = 67; 3+disorders, *N* = 24.

Adolescents diagnosed with more mental disorders had a more severe syndrome of despair-related maladies overall [*β* = 0.26, 95% CI (0.19–0.33), *p* < 0.001] and individually – suicidality [*β* = 0.26, 95% CI (0.19–0.33), *p* < 0.001], substance misuse [*β* = 0.19, 95% CI (0.11–0.26), *p* < 0.001], sleep problems [*β* = 0.21, 95% CI (0.14–0.28), *p* < 0.001], and pain [*β* = 0.19, 95% CI (0.12–0.29), *p* < 0.001], after adjusting for sex, childhood SES, and childhood IQ; Fig. 3, and see online Supplementary eTable 6 for unadjusted estimates).

### Associations between different types of adolescent mental disorder and the midlife despair syndrome

Adolescents who had an internalizing disorder [i.e. depression or an anxiety disorder; *β* = 0.14, 95% CI (0.07–0.22), *p* < 0.001] and adolescents who had an externalizing disorder [i.e. ADHD or conduct disorder; *β* = 0.24, 95% CI (0.16–0.30), *p* < 0.001] had more despair-related maladies overall at midlife, after adjusting for sex, childhood SES, and childhood IQ. Adolescents with an internalizing or externalizing disorder also had higher levels of each individual despair-related malady at midlife (Table 2). To test for unique effects of internalizing *v.* externalizing disorders, we also entered both variables into the model as simultaneous predictors. Both internalizing [*β* = 0.11, 95% CI (0.04–0.17), *p* < 0.001] and externalizing [*β* = 0.22, 95% CI (0.15–0.29), *p* < 0.001] disorders uniquely predicted higher levels of despair-related maladies overall after adjusting for sex, childhood SES, and childhood IQ, as well as higher levels of nearly every individual despair-related malady at midlife (see online Supplementary eTable 7).

**Table 2.** Each type of adolescent mental disorder was associated with a syndrome of midlife despair

Adolescent mental disorders	Associations with the midlife despair syndrome and its constituent elements				
	Despair factor	Suicidality subfactor	Substance misuse subfactor	Sleep problems subfactor	Pain subfactor
	*β* (95% CI)	*β* (95% CI)	*β* (95% CI)	*β* (95% CI)	*β* (95% CI)
Internalizing disorder	0.14* (0.07–0.22)	0.15* (0.07–0.22)	0.08* (0.01–0.15)	0.11* (0.04–0.18)	0.16* (0.08–0.24)
Depression	0.11* (0.03–0.19)	0.11* (0.04–0.20)	0.06 (−0.01 to 0.13)	0.09* (0.02–0.16)	0.10* (0.01–0.18)
Anxiety disorder	0.15* (0.08–0.22)	0.15* (0.08–0.23)	0.08* (0.01–0.17)	0.12* (0.05–0.19)	0.17* (0.10–0.25)
Externalizing Disorder	0.24* (0.16–0.30)	0.24* (0.16–0.32)	0.22* (0.14–0.30)	0.20* (0.13–0.27)	0.12* (0.05–0.20)
ADHD	0.10* (0.03–0.18)	0.10* (0.03–0.18)	0.04 (−0.03 to 0.12)	0.11* (0.04–0.19)	0.08* (0.00–0.17)
Conduct disorder	0.23* (0.16–0.31)	0.24* (0.16–0.31)	0.23* (0.15–0.31)	0.19* (0.12–0.26)	0.11* (0.04–0.18)

*Note.* Results displayed are adjusted estimates after adjusting for sex, childhood socioeconomic status, and childhood IQ. CI, confidence interval; ADHD, attention-deficit/hyperactivity disorder. * denotes *p* < 0.05.

Within the internalizing and externalizing categories, every individual adolescent mental disorder (depression and anxiety disorder; ADHD and conduct disorder) was associated with more despair-related maladies overall and individually at midlife. The sole exception to this pattern was that adolescent depression was not significantly associated with midlife substance misuse (Table 2). All associations remained significant even after adjusting for sex, childhood SES, and childhood IQ, except for the association between adolescent ADHD and midlife substance misuse.

### Is adolescent psychopathology associated with midlife despair above and beyond midlife psychopathology?

To address this question, we re-examined the association between adolescent psychopathology and the midlife despair factor after adjusting for age-45 psychopathology (online Supplementary eTable 8), operationalized in two ways: (1) the presence of any mental disorder (excluding alcohol and other substance use disorders, since these are already included in the midlife despair syndrome) at age 45, and (2) the number of mental disorders (again excluding alcohol and other substance use disorders) diagnosed at age 45. Adolescents diagnosed with a mental disorder were more likely to have a mental disorder at age 45 [OR 2.15, 95% CI (1.60–2.89), *p* < 0.001] and were diagnosed with more mental disorders at age 45 [*β* = 0.21, 95% CI (0.13–0.28), *p* < 0.001] than adolescents without a mental disorder. Even after adjusting for age-45 mental disorders, the association between adolescent psychopathology and the general midlife despair factor remained significant [*β* = 0.20, 95% CI (0.13–0.27), *p* < 0.001], beyond any age-45 mental disorder; *β* = 0.17, 95% CI (0.11–0.23), *p* < 0.001, beyond the sum of age-45 mental disorders; see online Supplementary eTable 8).

## Discussion

Findings from this longitudinal cohort study provide support for the hypothesis that individuals who exhibited more despair-related maladies at midlife, as captured by four interrelated subdomains – suicidality, substance misuse, sleep problems, and pain – tended to have had psychopathology in adolescence. Individuals with a more severe midlife despair syndrome also had more mental disorders as adolescents, and associations were not specific to any particular adolescent mental disorder or any particular subdomain of the midlife despair syndrome. Previous research suggests that psychopathology is associated with poorer health and diminished wellbeing later in life (Devendorf, Rum, Kashdan, & Rottenberg, 2022; Goodwin et al., 2009; Richmond-Rakerd, D'Souza, Milne, Caspi, & Moffitt, 2021; Scott et al., 2016). Our results extend previous findings in two ways. First, they provide a unified model for conceptualizing the interrelated maladies that comprise a midlife despair syndrome. Second, they trace the origins of the midlife despair syndrome back to psychopathology in adolescence, providing initial evidence for a robust early-life risk factor for multiple midlife despair-related maladies. Importantly, associations between adolescent psychopathology and the midlife despair syndrome could not be explained by other childhood risk factors, including deprived socioeconomic origins and low IQ.

The higher-order factor model of midlife despair-related maladies suggests that symptoms across four separate but interrelated domains – suicidality, substance misuse, sleep problems, and pain – coalesced to form a single overarching despair factor at midlife, which may represent a desire to escape from psychic and physical suffering. The roles of suicidality and substance misuse in causing deaths of despair (via suicides, drug overdoses, and alcohol-related liver disease) are already recognized. Indeed, based on the finding that the suicidality subfactor loaded so strongly (i.e. 0.96) on the general despair factor at midlife, it may be that suicidality represents the ultimate expression of despair. However, our findings suggest that sleep problems and pain should also be considered part of the constellation of despair-related maladies. Both are associated with decrements in functioning (Briggs et al., 2016; Stranges, Tigbe, Gómez-Olivé, Thorogood, & Kandala, 2012; Zelaya, Dahlhamer, Lucas, & Connor, 2020) and increased mortality (Grandner, Hale, Moore, & Patel, 2010; Smith, Wilkie, Croft, Parmar, & McBeth, 2018) as individuals age, and our findings suggest that they may contribute indirectly to mortality through their associations with suicidality and substance misuse (Case & Deaton, 2021; Chakravorty, Chaudhary, & Brower, 2016; Harris, Huang, Linthicum, Bryen, & Ribeiro, 2020; Valentino & Volkow, 2020). Clinically, our findings suggest that sleep problems and pain should be included in comprehensive assessments of mental health in midlife patients. Relatedly, midlife adults presenting for treatment at a mental health clinic, substance abuse clinic, sleep clinic, or pain clinic are likely to have other despair-related maladies in addition to their presenting concern, highlighting the need for coordinated interprofessional team-based care (Holmboe, Singer, Chappell, Assadi, Salman, & the Education & Training Working Group of the National Academy of Medicine; Action Collaborative on Countering the U.S. Opioid Epidemic, 2022).

Results support the generality (rather than specificity) of associations between adolescent psychopathology and the midlife despair syndrome, consistent with previous research suggesting that psychopathology confers non-specific risk for poorer aging-related outcomes at midlife (Wertz et al., 2021). Yet despite the pattern suggesting that it does not matter *which* mental disorder one has, having *more* comorbid adolescent mental disorders appeared to confer greater risk for the midlife despair syndrome. This pattern is consistent with previous research suggesting accumulative effects of psychopathology on individual despair-related maladies (Stone, Vander Stoep, & McCauley, 2016; Verona, Sachs-Ericsson, & Joiner, 2004).

Importantly, associations between adolescent psychopathology and the midlife despair syndrome were not explained away after adjusting for other factors known to increase risk for despair-related maladies (i.e. male sex, more deprived socioeconomic origins, lower childhood IQ). Associations were attenuated after adjusting for age-45 psychopathology, but remained significant, suggesting that the midlife despair syndrome is not redundant with midlife psychopathology in general, and observed associations do not merely reflect the continuity of psychopathology across the lifecourse. In addition to being a robust predictor, adolescent psychopathology also outperformed other potential risk factors in forecasting the midlife despair syndrome, with effect sizes multiple times as large (Fig. 2). These robust and relatively large effects provide support for the relevance of the observed associations, particularly given recent increases in the rates of childhood mental health problems globally (Racine et al., 2021) and calls to address the burgeoning youth mental health crisis (American Academy of Pediatrics, 2021; Office of the Surgeon General (OSG), 2021). Left unaddressed, this crisis may portend a swelling plague of midlife despair – and its attendant disability and mortality – in the coming decades.

Our findings have implications for future research, prevention, and health policy. First, our findings provide a foundation for future research examining the mechanisms linking adolescent psychopathology to a syndrome of midlife despair. Possible mechanisms include biological (e.g. common genetic vulnerability), psychological (e.g. stressful life experiences), social (e.g. erosion of social connections), educational/occupational (e.g. curtailed educational attainment), and lifestyle (e.g. poor health behaviors) factors. Second, if the association between adolescent psychopathology and the midlife despair syndrome turns out to be causal, prevention and treatment of psychopathology early in life has the potential to mitigate despair-related maladies and reduce deaths of despair (Deković et al., 2011; Swan et al., 2018). Third, even if adolescent psychopathology is merely a marker (rather than a true cause) of vulnerability for the midlife despair syndrome, policies are needed that protect this vulnerable population by prioritizing them to receive education and services.

This study has several limitations. First, the participants self-identified as predominantly White and originated only from New Zealand. Although New Zealand has not appeared to experience the same rise in deaths of despair as the U.S., mortality rates and deaths of despair in New Zealand show the same demographic patterning as in the U.S. – that is, they tend to cluster within more disadvantaged and less educated people (Blakely et al., 2008; Richmond-Rakerd, D'Souza, Milne, & Andersen, under review). Moreover, although income inequality is not as high in New Zealand as in the U.S., New Zealand still ranks 13th highest among the 38 OECD countries (8 places below the U.S. and 5 below the U.K.; OECD, 2022). Although the Dunedin Study has a good track record for replication of findings across a variety of domains [e.g. crime, personality, and psychopathology (Caspi et al., 1994); parenting behavior (Wertz et al., in press); social mobility (Belsky et al., 2018)], it is unknown whether the present study's findings will replicate in other geographic regions or ethnic groups. Second, participants who had died by suicide well before midlife (i.e. before age 26; *N* = 2) were not included in any analyses because we did not want to count suicides that occurred well before midlife as evidence of midlife despair. Third, this study was observational and thus cannot establish whether observed associations are causal. Future studies are necessary to ascertain whether the observed associations between adolescent psychopathology and the midlife despair syndrome are modifiable through mental health treatment. Fourth, we were unable to test a longitudinal path model from psychopathology to educational failure to difficult working conditions to pain and sleep problems to prescription medication to the despair syndrome including substance misuse, because our study lacked measures in the hypothesized sequential order (and power); however, we hope these findings will inspire researchers to test the implied causal model. Finally, we measured indicators of despair-related maladies but not actual deaths. Therefore, establishing direct connections to deaths of despair would require additional study and replication using other data sources. These limitations should be considered in light of the study's strengths, which include the use of measures of despair across multiple modalities, the decades-long timeframe of investigation, and the setting in New Zealand (allowing us to disentangle despair-related maladies from the opioid epidemic experienced in other countries such as the U.S.).

In conclusion, the findings of this cohort study suggest that individuals who exhibited a more severe syndrome of despair at midlife tended to have had a mental disorder in adolescence. Prevention and treatment of adolescent psychopathology could reduce vulnerability to the midlife despair syndrome and thereby lengthen the healthspans and lifespans of aging adults.

## Supporting information

Brennan et al. supplementary material Brennan et al. supplementary material

## Supplementary material

The supplementary material for this article can be found at https://doi.org/10.1017/S0033291723001320
