# Supplementary material for: Tracing the origins of midlife despair: association of psychopathology during adolescence with a syndrome of despair-related maladies at midlife
Source: Psychol Med. 2023 May 10;53(16):7569–80. doi: 10.1017/S0033291723001320 (PMC10636241; doi:10.1017/S0033291723001320)
Supplement: Brennan et al. supplementary material [file S0033291723001320sup001.docx]

**Supplementary Online Content**

**eAppendix 1.** Attrition Analysis

**eAppendix 2.** Confirmatory Factor Analysis Models

**eAppendix 3.** Construct Validation of the Midlife Despair Factor

**eAppendix 4.** Comparing Associations Between Adolescent Psychopathology and Despair-related Maladies Using Latent Factors in Structural Equation Modeling Versus Extracted Factor Scores

**eAppendix 5.** Sensitivity Analyses Including Individuals Who Died by Suicide

**eTable 1.** Standardized Factor Loadings for Models of Midlife Despair-related Maladies

**eTable 2.** Correlations Between Indicators of Midlife Despair-related Maladies

**eTable 3.** Associations Between Adolescent Psychopathology, Sex, Childhood SES, Childhood IQ, and Midlife Despair

**eTable 4.** Associations Between Adolescent Psychopathology and Observed Despair Indicators

**eTable 5.** Interactions Between Adolescent Psychopathology and Sex in the Prediction of Midlife Despair

**eTable 6.** Unadjusted Estimates for Associations Between Number of Adolescent Mental Disorders and Midlife Despair

**eTable 7.** Unique Associations Between Internalizing/Externalizing Disorders and Midlife Despair

**eTable 8.** Associations Between Adolescent Psychopathology and Midlife Despair-related Maladies After Adjusting for Age-45 Mental Disorders

**eFigure 1.** The Structure of Midlife Despair-related Maladies, Including Variances and Residuals

**eFigure 2.** Correlated Factors Model of the Structure of Midlife Despair

**eAppendix 1: Attrition Analysis**

We conducted an attrition analysis using childhood SES, childhood IQ, and history of psychopathology to determine whether participants in the Phase 45 data collection were representative of the original cohort.

No significant differences in childhood SES were found between the full cohort, those deceased, those still alive, or those seen at Phase 45.

No significant differences in childhood IQ were found between the full cohort, those still alive, or those seen at Phase 45. Those who were deceased by the Phase 45 data collection had significantly lower childhood IQ than those who were still alive (*t* = 2.09, *p* = .04).

No significant differences in history of psychopathology were found between the full cohort, those still alive, or those seen at Phase 45. Those who were deceased by the Phase 45 data collection had significantly more extensive histories of psychopathology (i.e.,higher p-factor scores) than those who were still alive (*t* = -2.86, *p* = .004).

**eAppendix 2: Confirmatory Factor Analysis Models**

Confirmatory factor analysis (CFA) was used to model the structure of midlife despair-related maladies. In CFA, latent continuous factors are hypothesized to account for the pattern of covariance among observed variables. Statistical analyses were performed using the weighted least squares means and variance adjusted (WLSMV) algorithm and 1,000 bootstrapped samples.(Muthén & Muthén, 1998-2017) The WLSMV estimator is appropriate for categorical and nonmultivariate normal data and provides consistent estimates when data are missing at random with respect to covariates.(Asparouhov & Muthén, 2010) The following variables were treated as categorical in analyses: suicide attempted, suicidality treatment, substance misuse treatment, sleep aid use, and pain medication use.

Using CFA, we tested two competing models of despair-related maladies at midlife—a higher-order factor model and a correlated-factors model. The higher-order factor model tests the hypothesis that there is a general despair factor, which influences four subfactors (i.e.,suicidality, sleep, substance misuse, and pain), each of which in turn influences a subset of the observed indicators. For example, pain medication use (an observed indicator) loads on the pain subfactor, which in turns loads on the general despair factor. The correlated-factors model tests the hypothesis that there are four correlated factors (i.e.,suicidality, substance misuse, sleep problems, and pain), each of which influences a subset of the observed indicators.

We assessed model fit using the comparative fit index (CFI), Tucker-Lewis index (TLI), root mean square error of approximation (RMSEA), and standardized root mean square residual (SRMR), compared against the following thresholds for establishing good fit: CFI≥.95, TLI≥.95, RMSEA≤.05, and SRMR≤.08.(Hu & Bentler, 1999; MacCallum, Browne, & Sugawara, 1996)

Results of the CFA indicated that the higher-order factor model (**Figure 1**, eFigure 1) fit the data closely: CFI=.96, TLI=.95, RMSEA=.04, 90% CI [.03, 0.05], SRMR=.07. Loadings for the suicidality, substance misuse, sleep problems, and pain subfactors on the general midlife despair factor were all significant and positive, ranging from .32 (pain) to .96 (suicidality). Loadings for all of the indicators on their respective subfactors were significant and positive, ranging from relatively weak (.11 for social jetlag) to strong (.94 for sleep aid use). Despite the relatively weak factor loading observed for social jetlag, we opted to retain this observed indicator in the model of midlife despair for both theoretical and empirical reasons: (1) social jetlag captures a unique component of the sleep problems construct; (2) it is clinically significant in that it represents a core feature of circadian rhythm sleep-wake disorders (American Psychiatric Association, 2013); (3) it is linked to important physical and mental health outcomes (e.g., Chaput et al., 2022; Levandovski et al., 2011); (4) it is inherent to jobs commonly held by adults considered to be at higher risk for deaths of despair (e.g., shift work); (5) removing social jetlag from the model resulted in an increase in the SRMR (a fit index that does not penalize model complexity) to .10, which is above the recommended threshold of .08; and (6) it is generally recommended that latent factors be estimated using at least three observed variables.

The correlated-factors model (eFigure 2) also fit the data closely: CFI=.96, TLI=.94, RMSEA=.04, 90% CI [0.03, 0.05], SRMR=.07. Loadings on the four factors, as well as correlations between the four factors, were all significant and positive (see eTable 1 for full results of both models; see eTable 2 for correlations among the observed indicators of midlife despair-related maladies). Because the higher-order factor model and the correlated-factors model are not nested, we could not directly compare them. Moreover, the WLSMV estimator does not produce comparative model fit indices such as the Akaike information criterion or Bayesian information criterion. We can conclude that both models fit our data similarly well, with the higher-order factor model offering a slightly more parsimonious solution. Thus, the higher-order factor model, which includes a general despair factor, was retained as the preferred model for accounting for individual differences in midlife despair-related maladies.

**eAppendix 3: Construct Validation of the Midlife Despair Factor**

Whereas the midlife despair syndrome includes symptoms of common behavioral health problems that are diagnosed and treated in clinical settings (e.g., a pain clinic), for construct validation analyses we selected variables that are conceptualized as related but external to the midlife despair syndrome. These external variables included demographic, social, and work-related factors, as well as general measures of subjective well-being (e.g., life satisfaction, perceived stress). These variables were all measured at age 45.

Although we considered including depression (and possibly anxiety) symptoms in the syndrome, we did not include symptoms of these or other psychiatric disorders for several reasons. First, based on previous research, it is not clear that these symptoms are truly part of the midlife despair syndrome (Copeland et al., 2020). Second, we wanted to avoid overlap between the midlife despair syndrome and adolescent psychopathology. Since depression and anxiety were included in the adolescent psychopathology variable, we wanted to be sure that the midlife despair syndrome was not simply a continued manifestation of adolescent psychopathology symptoms. This was a conservative and rigorous approach designed to make it more difficult to detect an association between adolescent psychopathology and the midlife despair syndrome. In other words, we wanted to minimize the potential for alternative explanations as to why there might be an association between adolescent psychopathology and a midlife despair syndrome. Thus, although we find it important to show that the midlife despair syndrome is related to depressive symptoms at midlife (see below), depression is not conceptualized as a necessary component of the syndrome.

| **Correlations between Continuous Variables and Midlife Despair Factor** | | |
| --- | --- | --- |
| **Construct** | ***r* (*p*)** | |
| ***Wellbeing, social connection, and stress*** |  | |
| Life satisfaction ^a^ | -.38* (<.001) | |
| Self-rated health | -.28* (<.001) | |
| Loneliness ^b^ | .26* (<.001) | |
| Social support ^c^ | -.21* (<.001) | |
| Perceived stress ^d^ | .37* (<.001) | |
| ***Finances and work*** |  | |
| Self-rated financial security/stability | -.30* (<.001) | |
| Occupational status/prestige ^e^ | -.18* (<.001) | |
| Physically demanding job | .20* (<.001) | |
| Pain and fatigue caused by work | .23* (<.001) | |
| Work cognitively demanding/stimulating | -.12* (<.001) | |
|  | | |
| **t-test Results for Associations between Categorical Variables and Midlife Despair Factor** | | |
| **Construct** | ***t* (*p*)** | **Mean Despair Factor Z-Score** |
| ***Hopefulness and mental health*** |  |  |
| Positive attitude toward aging ^f^ | 4.70* (<.001) |  |
| No |  | 0.11 |
| Yes |  | -0.19 |
| Reporting being “very likely” to live to age 75 | 6.20* (<.001) |  |
| No |  | 0.24 |
| Yes |  | -0.17 |
| DSM-based depression diagnosis | -9.93* (<.001) |  |
| No |  | -0.14 |
| Yes |  | 0.72 |
| Depressed mood for at least 2 weeks straight in the past year | -7.28* (<.001) |  |
| Absent (0) |  | -0.14 |
| Present (1 or 2) |  | 0.65 |
| Anhedonia for at least 2 weeks straight in the past year | -7.80* (<.001) |  |
| Absent (0) |  | -0.15 |
| Present (1 or 2) |  | 0.74 |
| ***Coping strategies*** ^g^ |  |  |
| Smoke more | -8.74* (<.001) |  |
| Not true |  | -0.17 |
| Somewhat true or very true |  | 0.71 |
| Drink more | -5.64* (<.001) |  |
| Not true |  | -0.15 |
| Somewhat true or very true |  | 0.22 |
| Obsess about problems | -4.78* (<.001) |  |
| Not true |  | -0.17 |
| Somewhat true or very true |  | 0.14 |
| Give up | -4.17* (<.001) |  |
| Not true |  | -0.08 |
| Somewhat true or very true |  | 0.41 |
| Sleep more | -2.75* (.007) |  |
| Not true |  | -0.06 |
| Somewhat true or very true |  | 0.21 |
| Ignore problems | -2.84* (.005) |  |
| Not true |  | -0.11 |
| Somewhat true or very true |  | 0.07 |
| Focus on other things (distract) | -2.89* (.004) |  |
| Not true |  | -0.18 |
| Somewhat true or very true |  | 0.04 |
| Take steps to solve problems | 2.46* (.017) |  |
| Not true |  | 0.43 |
| Somewhat true or very true |  | -0.04 |
| Withdraw more | -2.33* (.020) |  |
| Not true |  | -0.10 |
| Somewhat true or very true |  | 0.05 |
| Talk to others | 1.13 (.257) |  |
| Not true |  | 0.06 |
| Somewhat true or very true |  | -0.03 |
| Exercise more | 1.40 (.162) |  |
| Not true |  | 0.03 |
| Somewhat true or very true |  | -0.06 |
| Talk to a counselor | -0.89 (.372) |  |
| Not true |  | -0.03 |
| Somewhat true or very true |  | 0.05 |
| Eat more | 0.45 (.654) |  |
| Not true |  | 0.00 |
| Somewhat true or very true |  | -0.03 |
| Work more/harder | 0.71 (.476) |  |
| Not true |  | 0.04 |
| Somewhat true or very true |  | -0.02 |
| Pray or talk to a minister | -0.24 (.809) |  |
| Not true |  | -0.02 |
| Somewhat true or very true |  | 0.01 |
| ***Education and Work*** |  |  |
| Received Bachelor’s degree | 4.52* (<.001) |  |
| No |  | 0.09 |
| Yes |  | -0.20 |
| Work night shifts | -3.07* (.002) |  |
| No |  | -0.08 |
| Yes |  | 0.19 |
| Need stimulants to stay awake at work | -4.50* (<.001) |  |
| No |  | -0.11 |
| Yes |  | 0.32 |
| Will be physically unable to continue doing job in the future | -5.04* (<.001) |  |
| No |  | -0.13 |
| Yes |  | 0.30 |
| Unemployed for ≥1 month in past year | -1.97 (.058) |  |
| No |  | -0.02 |
| Yes |  | 0.51 |

* denotes *p* < .05

^a^ Satisfaction with Life Scale (Pavot & Diener, 2009)

^b^ UCLA Loneliness Scale (Russell, 1996)

^c^ Multidimensional Scale of Perceived Social Support (Zimet, Dahlem, Zimet, & Farley, 1988)

^d^ Perceived Stress Scale (Cohen, Kamarck, & Mermelstein, 1983)

^e^ Based on McMillan, Beavis, and Jones (2009)

^f^ Attitudes Toward Own Aging subscale of the Philadelphia Geriatric Center Morale Scale (Levy, Slade, Kunkel, & Kasl, 2002)

^g^ Described in Wertz et al. (2021)

**eAppendix 4: Comparing Associations Between Adolescent Psychopathology and Despair-related Maladies Using Latent Factors in Structural Equation Modeling Versus Extracted Factor Scores**

Regression analyses used midlife despair factor scores extracted from Mplus. Some readers may wish to see these associations using latent factors in the structural equation modeling framework. In the table below, we compare associations between adolescent psychopathology variables and midlife despair variables using extracted factor scores versus latent factors within the Mplus structural equation modeling environment. Results indicated similar patterns across both approaches; all associations estimated using extracted factor scores fell within the 95% confidence intervals of associations estimated using latent factors in Mplus.

|  |  | **Regression with extracted factor scores** | **Regression within structural equation modeling environment** |
| --- | --- | --- | --- |
| **Independent variable** | **Dependent variable** |  |  |
| ***Adolescent psychopathology*** |  |  |  |
|  | General despair factor | .25* (.18, .32) | .35* (.25, .44) |
|  | Suicidality subfactor | .25* (.19, .32) | .37* (.25, .50) |
|  | Substance misuse subfactor | .21* (.14, .28) | .22* (.14, .30) |
|  | Sleep problems subfactor | .20* (.13, .27) | .19* (.10, .27) |
|  | Pain subfactor | .19* (.12, .26) | .22* (.14, .32) |
| ***Number of adolescent mental disorders*** |  |  |  |
|  | General despair factor | .27* (.21, .35) | .37* (.27, .46) |
|  | Suicidality subfactor | .28* (.21, .35) | .39* (.27, .51) |
|  | Substance misuse subfactor | .20* (.14, .28) | .21* (.13, .29) |
|  | Sleep problems subfactor | .22* (.15, .29) | .22* (.12, .31) |
|  | Pain subfactor | .20* (.12, .29) | .24* (.14, .34) |
| ***Individual adolescent mental disorders*** |  |  |  |
| **Internalizing disorder** |  |  |  |
|  | General despair factor | .15* (.08, .22) | .21* (.09, .32) |
|  | Suicidality subfactor | .15* (.08, .23) | .29* (.06, .41) |
|  | Substance misuse subfactor | .08* (.01, .16) | .07 (-.01, .16) |
|  | Sleep problems subfactor | .11* (.04, .18) | .11* (.02, .20) |
|  | Pain subfactor | .17* (.10, .25) | .22* (.12, .31) |
| **Depression** |  |  |  |
|  | General despair factor | .11* (.04, .19) | .17* (.03, .28) |
|  | Suicidality subfactor | .12* (.04, .21) | .22* (-.01, .40) |
|  | Substance misuse subfactor | .06 (-.01, .13) | .04 (-.04, .12) |
|  | Sleep problems subfactor | .09* (.02, .17) | .10* (.002, .20) |
|  | Pain subfactor | .10* (.02, .19) | .12* (.00, .24) |
| **Anxiety disorder** |  |  |  |
|  | General despair factor | .16* (.08, .23) | .22* (.10, .33) |
|  | Suicidality subfactor | .16* (.09, .24) | .28* (.01, .41) |
|  | Substance misuse subfactor | .08* (.03, .16) | .07 (-.02, .15) |
|  | Sleep problems subfactor | .13* (.06, .20) | .13* (.04, .21) |
|  | Pain subfactor | .19* (.11, .26) | .24* (.15, .33) |
| **Externalizing disorder** |  |  |  |
|  | General despair factor | .25* (.18, .33) | .36* (.24, .44) |
|  | Suicidality subfactor | .26* (.18, .33) | .31* (.08, .45) |
|  | Substance misuse subfactor | .25* (.17, .32) | .27* (.17, .36) |
|  | Sleep problems subfactor | .20* (.13, .27) | .20* (.09, .29) |
|  | Pain subfactor | .13* (.06, .20) | .14* (.04, .24) |
| **ADHD** |  |  |  |
|  | General despair factor | .13* (.06, .20) | .16* (.05, .26) |
|  | Suicidality subfactor | .13* (.06, .21) | .13 (-.17, .28) |
|  | Substance misuse subfactor | .07* (-.01, .14) | .07 (-.01, .16) |
|  | Sleep problems subfactor | .12* (.05, .19) | .14* (.05, .22) |
|  | Pain subfactor | .09* (.01, .17) | .10 (.00, .21) |
| **Conduct disorder** |  |  |  |
|  | General despair factor | .25* (.19, .33) | .36* (.24, .45) |
|  | Suicidality subfactor | .25* (.17, .33) | .32* (.09, .45) |
|  | Substance misuse subfactor | .26* (.18, .33) | .29* (.18, .38) |
|  | Sleep problems subfactor | .20* (.13, .27) | .19* (.09, .28) |
|  | Pain subfactor | .11* (.04, .18) | .12* (.02, .23) |

**eAppendix 5: Sensitivity Analyses Including Individuals Who Died By Suicide**

Because some adult participants had completed suicide before age 45, we wanted to ensure that the exclusion of these participants from analyses did not skew or bias our results. In total, seven participants completed suicide after age 26 and before age 45. Six of these participants had full data for adolescent psychopathology, childhood IQ, and childhood SES. In order to include these participants in analyses, we recoded their age-45 standardized midlife despair general factor scores from ‘missing’ to the sample maximum (i.e., 4.68). With these six participants’ data added to the sample (new *N* = 889), we then re-ran all analyses that used the standardized midlife despair general factor score as the dependent variable. Results of these analyses were basically identical to those obtained using the original full sample that excluded participants who had completed suicide before age 45, suggesting that the exclusion of participants who completed suicide as adults did not bias our results (see table below for direct comparisons of standardized regression coefficients).

| **Adolescent psychopathology variable** | **Sample with addition of adults who died by suicide (*N* = 889)** | **Original full sample (*N* = 883)** |
| --- | --- | --- |
| Any adolescent psychopathology | .26* | .25* |
|  |  |  |
| Number of adolescent mental disorders | .28* | .27* |
|  |  |  |
| Internalizing disorders | .15* | .15* |
|  |  |  |
| Anxiety disorder | .14* | .16* |
|  |  |  |
| Depression | .15* | .11* |
|  |  |  |
| Externalizing disorders | .28* | .25* |
|  |  |  |
| ADHD | .12* | .13* |
|  |  |  |
| Conduct disorder | .28* | .25* |
|  |  |  |

*Note.* Values represent standardized regression coefficients (β) for the associations between adolescent psychopathology variables and the midlife despair factor. Shaded cells denote primary regression analyses examining the presence of any adolescent psychopathology as a predictor of midlife despair. * denotes *p* < .05

**eTable 1: Standardized Factor Loadings for Models of Midlife Despair-related Maladies**

| **Model Fit Statistics** | | | | | | | | | |
| --- | --- | --- | --- | --- | --- | --- | --- | --- | --- |
|  | **Higher Order Factor Model** | | | | | **Correlated Factors Model** | | | |
| Chi-Square (WLSMV) | 112.39 | | | | | 110.64 | | | |
| Degrees of Freedom | 50 | | | | | 48 | | | |
| Chi-Square *P* value | <.001 | | | | | <.001 | | | |
| Comparative Fit Index (CFI) | .96 | | | | | .96 | | | |
| Tucker-Lewis Index (TLI) | .95 | | | | | .94 | | | |
| Root mean square error of approximation (RMSEA)  (90% CI) | .037  (.028, .046) | | | | | .038  (.028, .047) | | | |
| Standardized root mean square residual (SRMR) | .073 | | | | | .072 | | | |
| **Standardized Factor Loadings**  **(95% CI)** | | | | | | | | | |
|  | **Higher Order Factor Model** | | | | | **Correlated Factors Model** | | | |
| **Observed Indicators of Despair-related Maladies** | **Suicidality** | **Substance misuse** | **Sleep problems** | **Pain** | **Despair** | **Suicidality** | **Substance misuse** | **Sleep problems** | **Pain** |
| Suicide attempted | .70  (.57, .91) |  |  |  |  | .70  (.47, .88) |  |  |  |
| Treatment for suicidality | .82  (.51, 1.02) |  |  |  |  | .82  (.37, 1.00) |  |  |  |
| Informant-reported suicidality | .33  (.21, .47) |  |  |  |  | .33  (.20, .45) |  |  |  |
| SUD symptoms |  | .74  (.67, .82) |  |  |  |  | .74  (.67, .82) |  |  |
| Treatment for substance misuse |  | .57  (.45, .67) |  |  |  |  | .57  (.46, .67) |  |  |
| Informant-reported substance misuse |  | .73  (.64, .82) |  |  |  |  | .73  (.64, .82) |  |  |

| **Standardized Factor Loadings**  **(95% CI)** | | | | | | | | | | | | | | | | |
| --- | --- | --- | --- | --- | --- | --- | --- | --- | --- | --- | --- | --- | --- | --- | --- | --- |
|  | **Higher Order Factor Model** | | | | | | | | | **Correlated Factors Model** | | | | | | |
| **Observed Indicators of Despair-related Maladies** | **Suicidality** | | **Substance misuse** | | **Sleep problems** | | **Pain** | | **Despair** | **Suicidality** | **Substance misuse** | | **Sleep problems** | | **Pain** | |
| Poor sleep quality (PSQI) |  | |  | | .51  (.43, .58) | |  | |  |  |  | | .51  (.43, .59) | |  | |
| Sleep aid use |  | |  | | .94  (.83, 1.00) | |  | |  |  |  | | .94  (.82, 1.00) | |  | |
| Social jetlag |  | |  | | .11  (.02, .22) | |  | |  |  |  | | .11  (.002, .21) | |  | |
| Musculoskeletal pain composite |  | |  | |  | | .61  (.48, .75) | |  |  |  | |  | | .61  (.48, .74) | |
| Life spheres with musculoskeletal pain interference |  | |  | |  | | .60  (.46, .71) | |  |  |  | |  | | .59  (.47, .71) | |
| Pain medication use |  | |  | |  | | .55  (.40, .75) | |  |  |  | |  | | .55  (.39, .72) | |
| **Despair subfactors** |  | |  | |  | |  | |  |  |  | |  | |  | |
| Suicidality subfactor |  | |  | |  | |  | | .96  (.70, .99) |  |  | |  | |  | |
| Substance misuse subfactor |  | |  | |  | |  | | .59  (.47, .71) |  |  | |  | |  | |
| Sleep problems subfactor |  | |  | |  | |  | | .80  (.65, .96) |  |  | |  | |  | |
| Pain subfactor |  | |  | |  | |  | | .32  (.15, .45) |  |  | |  | |  | |
| **Subfactor Correlations**  **(95% CI)** | | | | | | | | | | | | | | | | |
|  | **Higher Order Factor Model** | | | | | | | | | **Correlated Factors Model** | | | | | | |
| Suicidality subfactor |  |  | |  | |  | |  | | – | |  | |  | |  |
| Substance misuse subfactor |  |  | |  | |  | |  | | .58  (.39, .83) | | – | |  | |  |
| Sleep problems subfactor |  |  | |  | |  | |  | | .73  (.51, .98) | | .47  (.36, .59) | | – | |  |
| Pain subfactor |  |  | |  | |  | |  | | .31  (.08, .53) | | .15  (.02, .27) | | .30  (.18, .41) | | – |

*Note.* WLSMV = weighted least squares means and variance adjusted; SUD = substance use disorder; PSQI = Pittsburgh Sleep Quality Index; CI = confidence intervals.

**eTable 2: Correlations Between Observed Indicators of Midlife Despair-related Maladies**

| **Domain** | **Indicator** | **1** | **2** | **3** | **4** | **5** | **6** | **7** | **8** | **9** | **10** | **11** | **12** |
| --- | --- | --- | --- | --- | --- | --- | --- | --- | --- | --- | --- | --- | --- |
| Suicidality | 1) Suicide attempted | – |  |  |  |  |  |  |  |  |  |  |  |
|  | 2) Treatment for suicidality | .26* | – |  |  |  |  |  |  |  |  |  |  |
|  | 3) Informant-reported suicidality | .25* | .27* | – |  |  |  |  |  |  |  |  |  |
| Substance misuse | 4) SUD symptoms | .19* | .26* | .13* | – |  |  |  |  |  |  |  |  |
|  | 5) Treatment for substance misuse | .18* | .19 | .10 | .43* | – |  |  |  |  |  |  |  |
|  | 6) Informant-reported substance misuse | .25* | .20* | .17* | .56* | .32* | – |  |  |  |  |  |  |
| Sleep problems | 7) Poor sleep quality (PSQI) | .19* | .15* | .16* | .16* | .13* | .10* | – |  |  |  |  |  |
|  | 8) Sleep aid use | .21* | .15* | .21* | .35* | .20* | .29* | .43* | – |  |  |  |  |
|  | 9) Social jetlag | .03 | .04 | -.02 | .06 | .02 | .07 | .05 | .07 | – |  |  |  |
| Pain | 10) Musculoskeletal pain composite | .07 | -.02 | -.02 | .04 | .00 | .04 | .08* | .11* | -.04 | – |  |  |
|  | 11) Life spheres with musculoskeletal pain interference | .16* | -.01 | .01 | .04 | .08 | .11* | .12* | .12* | .01 | .42* | – |  |
|  | 12) Pain medication use^†^ | .17* | .08* | .04 | .05 | .01 | .11* | .13* | .12* | .04 | .28* | .18* | – |

*Note.* Correlations were calculated using MPlus. SUD = Substance use disorder. PSQI = Pittsburgh Sleep Quality Index. Shaded cells denote intra-domain correlations. * denotes *p* < .05

**eTable 3: Associations Between Adolescent Psychopathology, Sex, Childhood SES, Childhood IQ, and Midlife Despair**

| **Midlife despair and its constituent elements** | **Model** | **Predictor variables** | | | |
| --- | --- | --- | --- | --- | --- |
|  |  | **Adolescent psychopathology** | **Other risk factors** | | |
|  |  |  | **Male sex** | **Childhood SES** | **Childhood IQ** |
|  |  | **β (95% CI)** | **β (95% CI)** | **β (95% CI)** | **β (95% CI)** |
| Despair factor | Univariable | .25* (.18, .32) | .07* (.01, .14) | -.08* (-.15, -.02) | -.09* (-.15, -.03) |
|  | Multivariable | .23* (.16, .30) | .07* (.01, .13) | -.03 (-.10, .04) | -.05 (-.11, .02) |
| Suicidality  subfactor | Univariable | .25* (.19, .32) | .07* (.01, .14) | -.09* (-.15, -.03) | -.10* (-.15, -.04) |
|  | Multivariable | .24* (.17, .31) | .07* (.01, .13) | -.03 (-.10, .04) | -.05 (-.12, .02) |
| Substance  misuse subfactor | Univariable | .21* (.14, .28) | .15* (.09, .22) | -.07* (-.13, .00) | -.05 (-.11, .01) |
|  | Multivariable | .19* (.12, .27) | .15* (.08, .21) | -.03 (-.10, .03) | -.01 (-.08, .05) |
| Sleep problems  subfactor | Univariable | .20* (.13, .27) | .04 (-.03, .10) | -.05 (-.11, .02) | -.07* (-.13, -.001) |
|  | Multivariable | .19* (.12, .26) | .03 (-.03, .10) | .00 (-.07, .07) | -.04 (-.11, .03) |
| Pain subfactor | Univariable | .19* (.12, .26) | -.04 (-.10, .03) | -.09* (-.16, -.02) | -.09* (-.15, -.02) |
|  | Multivariable | .18* (.11, .25) | -.04 (-.10, .03) | -.04 (-.11, .04) | -.04 (-.11, .04) |

*Note.* The top values within each box correspond to the standardized regression coefficients (β) and bootstrapped 95% CIs for the respective univariable (simple) linear regression models; the bottom values within each box correspond to the standardized regression coefficients (β) and bootstrapped 95% CIs for the multivariable (multiple) linear regression model. Shaded cells denote primary regression analyses examining adolescent psychopathology as a predictor of midlife despair. CI = confidence interval. * denotes *p* < .05. The associations between adolescent psychopathology and midlife despair-related maladies survived a Bonferroni-corrected α of .01.

| **Suicidality** | | **Substance Misuse** | |
| --- | --- | --- | --- |
| **Observed Indicator** | **Correlation coefficient**  **(95% CI)** | **Observed Indicator** | **Correlation coefficient**  **(95% CI)** |
| Suicide attempted | .25*  (.17, .33) | SUD symptoms | .18*  (.09, .26) |
| Treatment for suicidality | .05  (-.05, .19) | Treatment for substance misuse | .16*  (.07, .50) |
| Informant-reported suicidality | .12*  (.03, .23) | Informant-reported substance misuse | .20*  (.11, .28) |
| **Sleep Problems** | | **Pain** | |
| **Observed Indicator** | **Correlation coefficient**  **(95% CI)** | **Observed Indicator** | **Correlation coefficient**  **(95% CI)** |
| Poor sleep quality (PSQI) | .14*  (.07, .22) | Musculoskeletal pain composite | .13*  (.04, .21) |
| Sleep aid use | .16*  (.07, .24) | Life spheres with musculoskeletal pain interference | .18*  (.09, .27) |
| Social jetlag | .12*  (.04, .21) | Pain medication use | .17*  (.08, .26) |

**eTable 4: Associations Between Adolescent Psychopathology and Observed Indicators of Midlife Suicidality, Sleep Problems, Substance Misuse, and Pain**

*Note.* Correlations were calculated using MPlus, with adolescent psychopathology treated as a categorical variable. CI = confidence interval; SUD = substance use disorder; PSQI = Pittsburgh Sleep Quality Index. * denotes *p* < .05

**eTable 5: No Moderation of Adolescent Psychopathology by Sex, Childhood SES, or Childhood IQ in the Prediction of the General Midlife Despair Factor**

|  | **Interaction term results** | | | | | |
| --- | --- | --- | --- | --- | --- | --- |
|  | **Interaction with sex** | | **Interaction with childhood SES** | | **Interaction with childhood IQ** | |
| **Adolescent psychopathology variable** | **Model including adolescent psychopathology, sex, and their interaction** | **Model adjusting for sex, childhood SES, and childhood IQ** | **Model including adolescent psychopathology, childhood SES, and their interaction** | **Model adjusting for sex, childhood SES, and childhood IQ** | **Model including adolescent psychopathology, childhood IQ, and their interaction** | **Model adjusting for sex, childhood SES, and childhood IQ** |
|  | ***F* (*p*) for the interaction** | ***F* (*p*) for the interaction** | ***F* (*p*) for the interaction** | ***F* (*p*) for the interaction** | ***F* (*p*) for the interaction** | ***F* (*p*) for the interaction** |
| Any adolescent psychopathology | 0.01 (.942) | 0.00 (1.000) | 0.35 (.553) | 0.27 (.607) | 2.85 (.092) | 2.47 (.117) |
| Number of adolescent mental disorders | 0.39 (.759) | 0.44 (.726) | 0.69 (.560) | 0.69 (.560) | 0.51 (.675) | 0.43 (.729) |
| Internalizing | 1.67 (.196) | 1.57 (.211) | 0.42 (.515) | 0.37 (.544) | 0.55 (.460) | 0.57 (.451) |
| Depression | 0.41 (.521) | 0.39 (.533) | 1.73 (.188) | 1.62 (.203) | 1.07 (.302) | 1.15 (.285) |
| Anxiety disorder | 1.80 (.180) | 1.77 (.183) | 0.40 (.529) | 0.33 (.569) | 0.21 (.645) | 0.19 (.664) |
| Externalizing | 1.73 (.189) | 1.67 (.197) | 0.25 (.620) | 0.25 (.621) | 0.44 (.506) | 0.41 (.524) |
| ADHD | 0.00 (.953) | 0.03 (869) | 0.10 (.757) | 0.06 (.812) | 0.41 (.521) | 0.55 (.458) |
| Conduct disorder | 2.85 (.092) | 2.66 (.104) | 0.83 (.362) | 0.83 (,363) | 0.04 (.835) | 0.02 (.890) |

**eTable 6: Unadjusted Estimates for Associations Between Number of Adolescent Mental Disorders and Midlife Despair**

| **Dependent variable** | **Number of adolescent mental disorders** |
| --- | --- |
|  |  |
| Despair factor | .27* (.21, .35) |
| Suicidality  subfactor | .28* (.21, .35) |
| Substance  misuse subfactor | .20* (.14, .28) |
| Sleep problems  subfactor | .22* (.15, .29) |
| Pain subfactor | .20* (.12, .29) |

*Note.* Values correspond to standardized regression coefficients (β) and bootstrapped 95% CIs. CI = confidence interval. * denotes *p* < .05

**eTable 7: Unique Associations Between Internalizing/Externalizing Disorders and Midlife Despair**

|  |  | **Unique predictions of internalizing/externalizing disorders** | |
| --- | --- | --- | --- |
| **Midlife despair and its constituent elements** | **Independent variable** | **Model with no other covariates** | **Model adjusting for sex, childhood IQ, and childhood SES** |
|  |  |  |  |
|  |  |  |  |
|  |  |  |  |
|  |  | **β (95% CI)** | **β (95% CI)** |
| Despair factor | Internalizing disorder | .11* (.04, .17) | .11* (.04, .17) |
|  | Externalizing disorder | .24* (.17, .30) | .22* (.15, .29) |
| Suicidality  subfactor | Internalizing disorder | .11* (.05, .18) | .11* (.05, .18) |
|  | Externalizing disorder | .24* (.17, .30) | .22* (.15, .29) |
| Substance  misuse subfactor | Internalizing disorder | .04 (-.03, .10) | .05 (-.02, .12) |
|  | Externalizing disorder | .24* (.18, .31) | .21* (.15, .28) |
| Sleep problems  subfactor | Internalizing disorder | .08* (.02, .15) | .08* (.01, .15) |
|  | Externalizing disorder | .19* (.12, .25) | .18* (.11, .25) |
| Pain subfactor | Internalizing disorder | .15* (.09, .22) | .14* (.08, .21) |
|  | Externalizing disorder | .11* (.04, .17) | .10* (.03, .17) |

*Note.* * denotes *p* < .05

**eTable 8: Associations Between Adolescent Psychopathology and Midlife Despair-related Maladies After Adjusting for Midlife Psychopathology**

|  |  | **Univariate associations between psychopathology and midlife despair** | | **Unique predictions of adolescent psychopathology and midlife psychopathology** | | **Unique predictions of adolescent psychopathology and midlife psychopathology after adjusting for sex, childhood SES, and childhood IQ** | |
| --- | --- | --- | --- | --- | --- | --- | --- |
| **Midlife despair and its constituent elements** | **Independent variable** | **Model with any mental disorder at midlife** | **Model with sum of mental disorders at midlife** | **Model with any mental disorder at midlife** | **Model with sum of mental disorders at midlife** | **Model with any mental disorder at midlife** | **Model with sum of mental disorders at midlife** |
|  |  | **β (95% CI)** | **β (95% CI)** | **β (95% CI)** | **β (95% CI)** | **β (95% CI)** | **β (95% CI)** |
| Despair factor | Adolescent psychopathology | .25* (.18, .32) | | .21* (.14, .28) | .18* (.13, .25) | .20* (.13, .27) | .17* (.11, .23) |
|  | Midlife psychopathology | .26* (.19, .34) | .31* (.23, .39) | .23* (.16, .30) | .28* (.20, .35) | .24* (.17, .32) | .28* (.20, .36) |
| Suicidality  subfactor | Adolescent psychopathology | .25* (.19, .32) | | .21* (.15, .28) | .19* (.12, .25) | .20* (.14, .27) | .17* (.11, .24) |
|  | Midlife psychopathology | .27* (.20, .35) | .32* (.24, .40) | .23* (.16, .31) | .28* (.20, .36) | .24* (.17, .31) | .29* (.21, .37) |
| Substance  misuse  subfactor | Adolescent psychopathology | .21* (.14, .28) | | .18* (.12, .25) | .16* (.09, .23) | .17* (.10, .24) | .15* (.08, .22) |
|  | Midlife psychopathology | .19* (.12, .27) | .22* (.13, .32) | .16* (.09, .24) | .19* (.10, .28) | .18* (.10, .26) | .21* (.12, .30) |
| Sleep  problems  subfactor | Adolescent psychopathology | .20* (.13, .27) | | .16* (.09, .22) | .14* (.07, .21) | .16* (.09, .23) | .14* (.07, .20) |
|  | Midlife psychopathology | .23* (.16, .30) | .28* (.20, .35) | .21* (.14, .28) | .25* (.17, .32) | .21* (.14, .28) | .25* (.18, .33) |
| Pain subfactor | Adolescent psychopathology | .19* (.12, .26) | | .17* (.10, .23) | .15* (.08, .21) | .16* (.09, .23) | .14* (.07, .21) |
|  | Midlife psychopathology | .16* (.09, .23) | .18* (.10, .26) | .13* (.06, .19) | .15* (.07, .23) | .12* (.06, .19) | .15* (.07, .22) |

*Note.* The midlife psychopathology variables included the following mental disorders assessed at age 45: any anxiety disorder (including generalized anxiety disorder, social anxiety disorder, agoraphobia, specific phobia, and panic disorder), obsessive-compulsive disorder, post-traumatic stress disorder, depression, mania, and psychotic disorder (possible range for sum of mental disorders: 0-6; Caspi et al., 2020); * denotes *p* < .05

**eFigure 1: The Structure of Midlife Despair-related Maladies, Including Variances and Residuals**

**
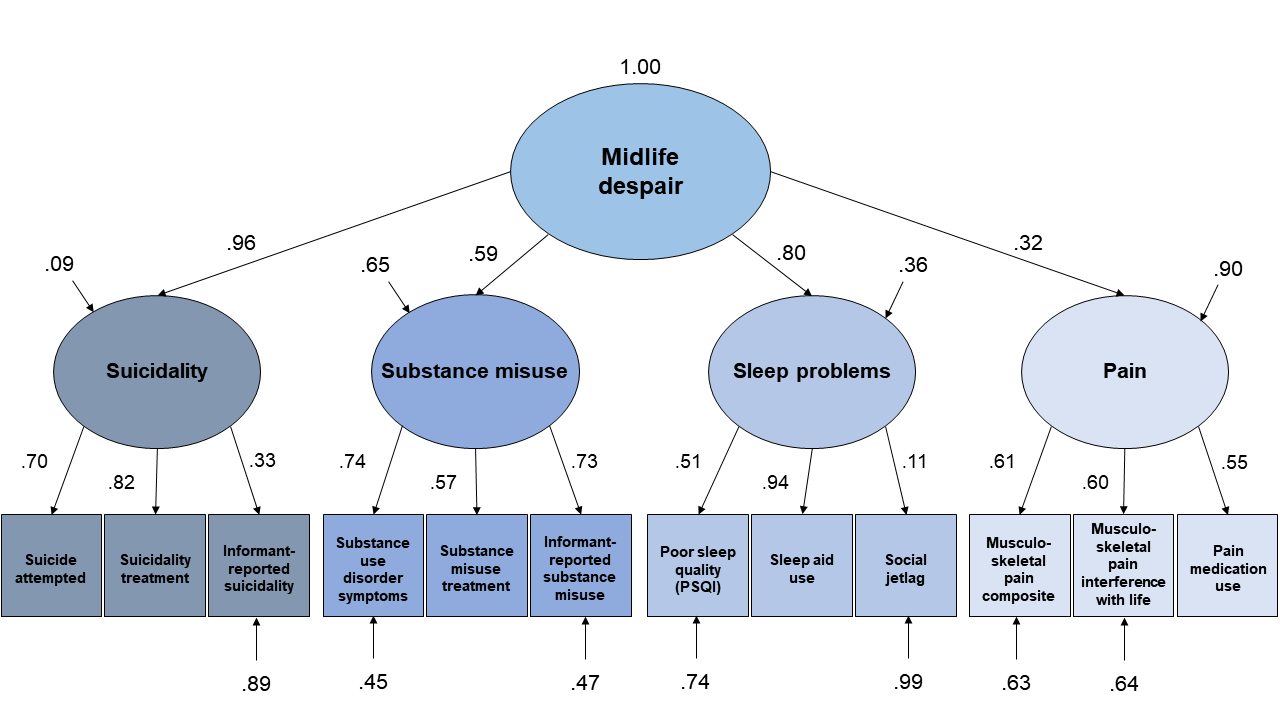
**

*Note.* Ovals are latent (unobserved) factors representing a syndrome of midlife despair and its constituent elements; boxes are observed indicators of each constituent element. Numbers are standardized factor loadings, variances, and residuals. Variables treated as categorical do not have residuals.

**eFigure 2: Correlated Factors Model of the Structure of Midlife Despair**


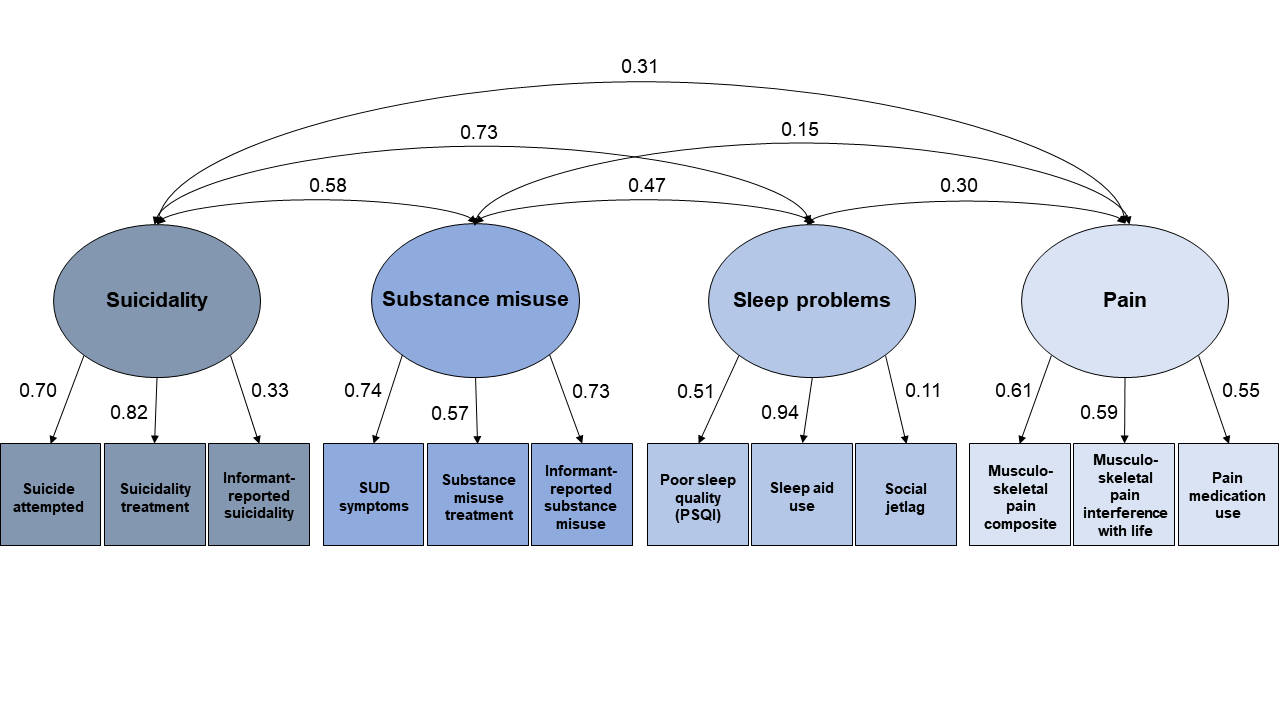


*Note.* Ovals represent latent (unobserved) factors representing midlife despair and its constituent elements; boxes represent observed indicators of each constituent element. Numbers represent standardized factor loadings (straight arrows) and correlation coefficients (curved arrows). SUD = substance use disorder; PSQI = Pittsburgh Sleep Quality Index.

**eReferences**

American Psychiatric Association. (2013). *Diagnostic and Statistical Manual of Mental Disorders* (5th ed.). Washington, DC: American Psychiatric Association.

Asparouhov, T., & Muthén, B. O. (2010). *Weighted least squares estimation with missing data*. Retrieved from <http://www.statmodel.com/download/GstrucMissingRevision.pdf>

Caspi, A., Houts, R. M., Ambler, A., Danese, A., Elliott, M. L., Hariri, A., . . . Moffitt, T. E. (2020). Longitudinal Assessment of Mental Health Disorders and Comorbidities Across 4 Decades Among Participants in the Dunedin Birth Cohort Study. *JAMA Network Open, 3*(4), e203221-e203221. doi:10.1001/jamanetworkopen.2020.3221

Chaput, J.-P., McHill, A. W., Cox, R. C., Broussard, J. L., Dutil, C., da Costa, B. G. G., . . . Wright, K. P. (2022). The role of insufficient sleep and circadian misalignment in obesity. *Nature Reviews Endocrinology*. doi:10.1038/s41574-022-00747-7

Cohen, S., Kamarck, T., & Mermelstein, R. (1983). A global measure of perceived stress. *Journal of Health and Social Behavior, 24*(4), 385-396.

Copeland, W. E., Gaydosh, L., Hill, S. N., Godwin, J., Harris, K. M., Costello, E. J., & Shanahan, L. (2020). Associations of despair with suicidality and substance misuse among young adults. *JAMA Network Open, 3*(6). doi:10.1001/jamanetworkopen.2020.8627

Hu, L. T., & Bentler, P. M. (1999). Cutoff criteria for fit indexes in covariance structure analysis: Conventional criteria versus new alternatives. *Structural Equation Modeling, 6*(1), 1-55. doi:10.1080/10705519909540118

Levandovski, R., Dantas, G., Fernandes, L. C., Caumo, W., Torres, I., Roenneberg, T., . . . Allebrandt, K. V. (2011). Depression scores associate with chronotype and social jetlag in a rural population. *Chronobiology International, 28*(9), 771-778. doi:10.3109/07420528.2011.602445

Levy, B. R., Slade, M. D., Kunkel, S. R., & Kasl, S. V. (2002). Longevity increased by positive self-perceptions of aging. *Journal of Personality and Social Psychology, 83*(2), 261.

MacCallum, R. C., Browne, M. W., & Sugawara, H. M. (1996). Power analysis and determination of sample size for covariance structure modeling. *Psychological Methods, 1*(2), 130-149. doi:10.1037/1082-989x.1.2.130

McMillan, J., Beavis, A., & Jones, F. L. (2009). The AUSEI06:A new socioeconomic index for Australia. *Journal of Sociology, 45*(2), 123-149. doi:10.1177/1440783309103342

Muthén, L. K., & Muthén, B. O. (1998-2017). *Mplus User's Guide* (8th ed.). Los Angeles, CA: Muthén & Muthén.

Pavot, W., & Diener, E. (2009). Review of the Satisfaction With Life Scale. In E. Diener (Ed.), *Assessing Well-Being: The Collected Works of Ed Diener* (pp. 101-117). Dordrecht: Springer Netherlands.

Russell, D. W. (1996). UCLA Loneliness Scale (Version 3): Reliability, validity, and factor structure. *Journal of Personality Assessment, 66*(1), 20-40. doi:10.1207/s15327752jpa6601_2

Wertz, J., Israel, S., Arseneault, L., Belsky, D. W., Bourassa, K. J., Harrington, H., . . . Caspi, A. (2021). Vital personality scores and healthy aging: Life-course associations and familial transmission. *Soc Sci Med, 285*, 114283. doi:10.1016/j.socscimed.2021.114283

Zimet, G. D., Dahlem, N. W., Zimet, S. G., & Farley, G. K. (1988). The Multidimensional Scale of Perceived Social Support. *Journal of Personality Assessment, 52*(1), 30-41. doi:10.1207/s15327752jpa5201_2
